# Supplementary material for: MARCKS Inhibition Alters Bovine Neutrophil Responses to Salmonella Typhimurium
Source: Biomedicines. 2024 Feb 16;12(2):442. doi: 10.3390/biomedicines12020442 (PMC10886653; doi:10.3390/biomedicines12020442)
Supplement: Supplementary file 1 [file biomedicines-12-00442-s001.zip › biomedicines-2821249-supplementary.pdf]

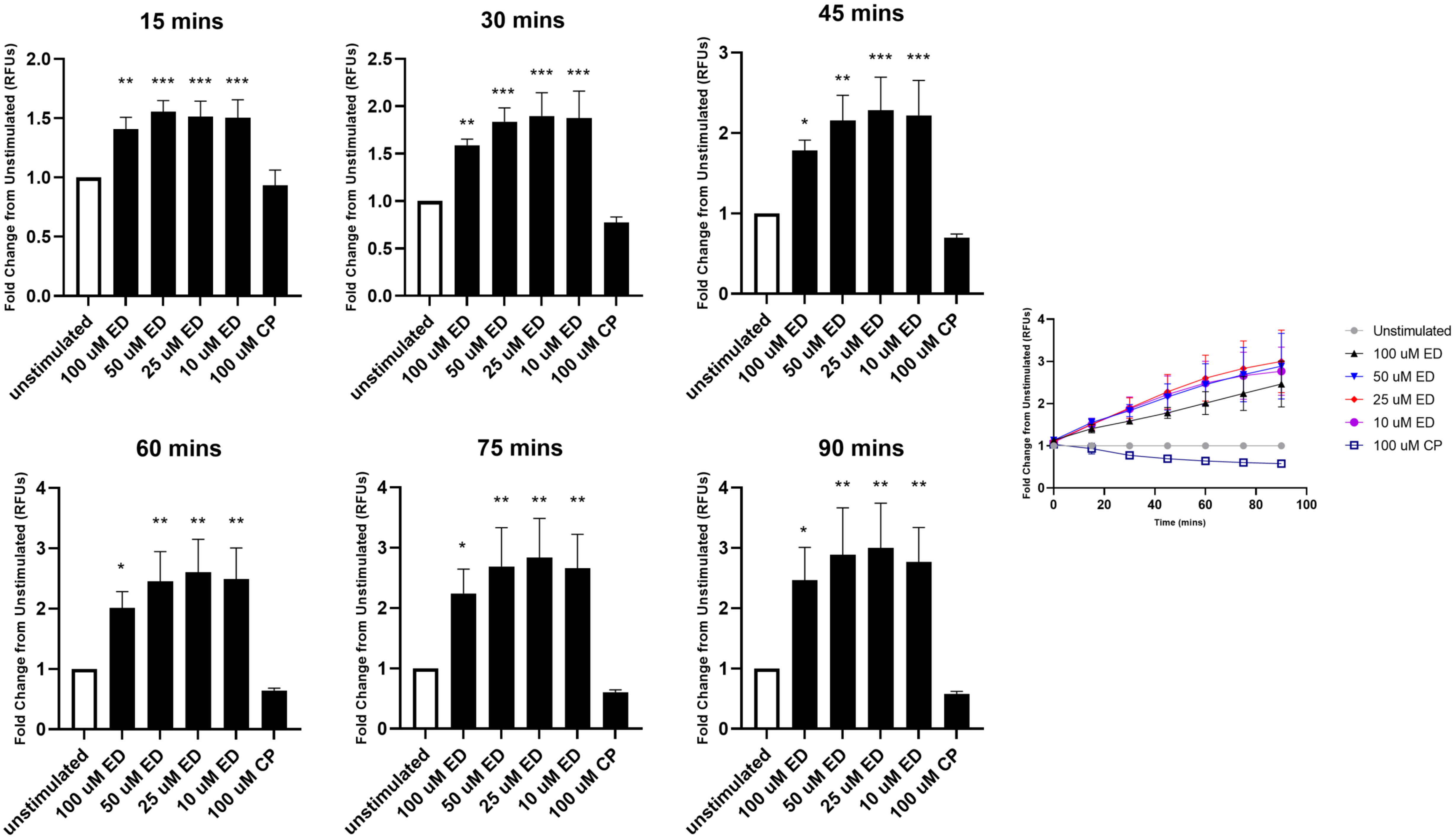

Supplemental Figure S1. ED peptide induces respiratory burst in primary bovine neutrophils. Isolated neutrophils were primed for 30 minutes with GM-CSF then treated with indicated concentrations of peptide for 30 minutes. Cells were plated in 96 well plate, DHR added, and fluorescence was read every 15 minutes for 90 minutes. Data represented as mean ± SD (n=3). Ordinary one-way ANOVA with Dunnett's multiple comparisons test. *p<0.05 vs unstimulated.
